# Pharmacokinetics and Pharmacological Activities of Berberine in Diabetes Mellitus Treatment

**DOI:** 10.1155/2021/9987097

**Published:** 2021-08-21

**Authors:** Yunfeng Han, Yunan Xiang, Yi Shi, Xi Tang, Lin Pan, Jie Gao, Ruohong Bi, Xianrong Lai

**Affiliations:** ^1^School of Pharmacy, Chengdu University of Traditional Chinese Medicine, Chengdu 611137, Sichuan, China; ^2^School of Ethnic Medicine, Chengdu University of Traditional Chinese Medicine, No. 1166 Liutai Avenue, Wenjiang District, Chengdu 611137, Sichuan, China

## Abstract

Traditional Chinese medicine (TCM) has good clinical application prospects in diabetes treatment. In addition, TCM is less toxic and/or has fewer side effects and provides various therapeutic effects. Berberine (BBR) is isolated as the main component in many TCM kinds (e.g., *Rhizoma Coptidis* and *Berberidis Cortex*). Furthermore, BBR can reduce blood sugar and blood fat, alleviate inflammation, and improve the state of patients. Based on the recent study results of BBR in diabetes treatment, the BBR pharmacokinetics and mechanism on diabetes are mainly studied, and the specific molecular mechanism of related experimental BBR is systematically summarized and analyzed. Clinical studies have proved that BBR has a good therapeutic effect on diabetes, suggesting that BBR may be a promising drug candidate for diabetes. More detailed BBR mechanisms and pathways of BBR need to be studied further in depth, which will help understand the BBR pharmacology in diabetes treatment.

## 1. Introduction

The prevalence of diabetes has shown a sharply rising trend in recent years and has become the ninth leading cause of mortality threatening human health. Although metformin and glucocorticoids have good curative effects, their application is often restricted due to some adverse reactions. Therefore, urgently finding safe and effective natural medicines with few side effects to prevent and treat diabetes is necessary. A large number of experiments have confirmed that berberine (BBR) is such a natural medicine. The natural product BBR is a type of isoquinoline alkaloid that has been used for diabetes treatment for more than 1,400 years in Chinese medical remedy history that is widely present in various botanicals/medicinal Chinese herbs (e.g., *Rhizoma Coptidis* and *Berberidis Cortex*). Various pharmacological experiments have been conducted to evaluate the effects of antidiabetic BBR and its complications in various cell and animal disease models with promising results.

However, the low BBR bioavailability and the unclear mechanism of action are the problems where its clinical application is greatly limited. This article aims to study the BBR pharmacokinetics and roughly explains the changes under the influence of the body after BBR administration to clarify the reasons for the BBR poor absorption and propose solutions to provide new formulations with high bioavailability in the future. Simultaneously, by summarizing the molecular mechanism of antidiabetic BBR, many pathway studies were noted to be relatively superficial. Thus, a more in-depth study is needed to determine its detailed mechanism to provide a reference for subsequent animal and human experimental designs [1–3].

## 2. Pharmacokinetic BBR Profile

### 2.1. Absorption

BBR has a good therapeutic effect on various diseases, but its clinical application is limited to a certain extent because of its poor oral absorption and low bioavailability [3, 4]. In terms of clinical medication, although intravenous injection can increase the drug concentration in the blood, clinical trials found that it will cause serious side effects (e.g., drop in blood pressure and respiratory arrest). Thus, oral administration is the main clinical BBR administration method. However, the absolute BBR bioavailability in rats is <1%. The poor absorption after BBR oral administration is caused by poor permeability, P-glycoprotein- (P-gp-) mediated efflux, hepatobiliary reexcretion, and self-aggregation. Moreover, the low BBR oral bioavailability may be due to its poor absorption and the first-pass effect in the liver as well as in the intestine [5].

Previous experiments have proved that the BBR bioavailability can be improved in various ways. On the one hand, altering the delivery system is a pretty good way to improve bioavailability. On the other hand, the BBR chemical structure modification, especially the synthesis of derivatives with an antidiabetic effect, is also a common approach for improving BBR's efficacy [6]. Additionally, some experiments [7] proved that the coadministration of BBR and P-gp inhibitors was also effective. The specific examples of the three aforementioned methods are shown in Table 1. Furthermore, Cui et al. found that the bioavailability of berberine organic acid salts, especially BBR fumarate and BBR succinate, is higher than that of BBR hydrochloride [8]. Therefore, fully considering the different BBR administration forms to fully exert the BBR pharmacological effects is necessary for the clinical application process.

### 2.2. Distribution

Some studies [27, 28] revealed that BBR is mainly distributed in tissues after oral administration with lower plasma content. After intragastric dosing, BBR was rapidly distributed in the liver, kidneys, muscle, lungs, brain, heart, pancreas, and fat in descending amount order. Thus, the liver is the main distribution organ. The pharmacokinetic profile indicated that the BBR level in most of the studied tissues was about 70 times higher than that in plasma 4 h after administration. However, it remained relatively stable in tissues (e.g., liver, muscle, brain, heart, and pancreas).

### 2.3. Metabolism

Numerous experimental studies have confirmed that BBR has a quick metabolic rate in vivo, which is mainly metabolized by the liver and intestinal flora. Moreover, most of it is metabolized into other components. Therefore, BBR content and structure will change accordingly after BBR is metabolized by the liver and intestinal flora. The major metabolic BBR pathways were demethylation, demethylenation, reduction, and hydroxylation (phase I BBR metabolites), and subsequent glucuronidation, sulfation, and methylation (phase II BBR metabolites) were the major metabolic BBR pathways in vivo [29–31].

Earlier studies have demonstrated that BBR metabolism is closely related to the cytochrome P450 (CYP450) enzymes in the liver [11]. Studies on BBR metabolism have shown that BBR can be metabolized by CYP450 enzyme subtypes, of which the most significant metabolic enzymes are CYP2D6 and CYP1A2, which play a crucial role in BBR biotransformation in mice and humans [32, 33]. After 0.5 h and 1 h after oral administration of BBR and original drug, the main metabolites and their respective glucuronic acid conjugates were detected in the liver tissues and bile, respectively [28].

The intestine is the main part of oral administration for absorption and metabolism. As an invisible “organ” in the body, the gut microbiota is the main participant. Previous studies have indicated that BBR undergoes a series of biotransformation processes in vivo after oral administration under the action of the gut microbiota, and its property and content have changed, which may be affected by the intestinal flora. Previous experiments [34, 35] have demonstrated that the main metabolites produced by BBR after the intestinal flora conversion are two types of demethoxy and hydrogenation. The BBR hydrogenation metabolic pathway is BBR can be metabolized by nitroreductases (NRs) produced by the intestinal flora into its absorbable form of dihydroberberine, which then oxidizes back to BBR after absorption in intestine tissues and enters the blood. Studies on this process are conducive to the more in-depth elucidation of the material basis of the drug's effect.

### 2.4. Excretion

BBR is mainly excreted in the kidneys (urine and feces) and bile in different animals. Bile excretion is slow due to the presence of hepatoenteric circulation. Experiments have proved that the different experimental subjects (rabbits, mice, rats, and humans) and different BBR administration methods (gavage, oral, and intravenous administration) will both cause excretion differences.

According to relevant experimental studies [36, 37], it is not difficult to find that, for rats, feces are the main excretion method during gavage or oral administration, and BBR in feces is mainly excreted in its original form. However, urine and bile excretion are the opposite. Their excretion is much lower than feces, and the excretion of BBR metabolites is greater than the prototype. Urine is the main excretion method when intravenously administered. For instance, BBR and many of its metabolites were quantitatively detected by high-performance liquid chromatography-tandem mass spectrometry. The total recovered BBR rate was 22.83%. The recovered rate of the prototype was more than fivefold that of its metabolites, while the BBR excretion in descending order was feces > urine > bile. Additionally, most BBR and its metabolites were found in the feces containing 84% of the prototype.

As for mice, the experiment [33] proved that BBR (I), the prototype administered, was the most abundant compound in urine, and urine excretion amount was about twofold that in feces. In addition, 11 metabolites were detected in feces and urine: six conjugated (metabolites III, V, VIII, IX, X, and XI) and five unconjugated (metabolites II, IV, VI, VII, and XII) metabolites.

## 3. Studies on the BBR Mechanism

**Figure 1:**
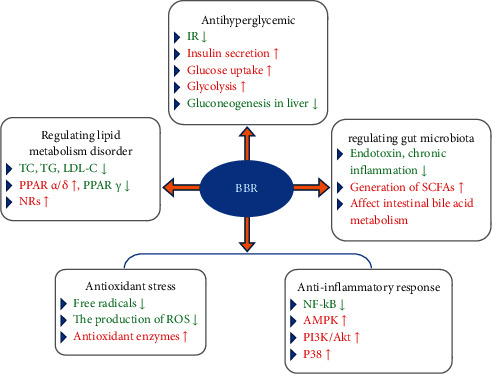
The various methods of BBR treatment of diabetes (↑ = increase or improve; ↓ = decrease or inhibit).

Streptozotocin (STZ) is toxic to *β*-cells and can be used to induce diabetes models. In this rat model, BBR shows significant *β*-cell regeneration, insulin secretion, insulin sensitization, and antioxidant activity [38]. Therefore, most of the various mechanisms described below are discussed based on the STZ-induced diabetic model. BBR has a good effect on metabolic syndrome such as diabetes [39–41]. It does not only lower blood sugar by enhancing insulin sensitivity, promoting insulin secretion, protecting islet cells, stimulating glycolysis in peripheral tissue cells, inhibiting gluconeogenesis in the liver, and anti-insulin resistance (IR) but also improves the symptoms of inflammation, oxidative stress, and lipid metabolism disorders in diabetic patients by anti-inflammatory, antioxidant, antiapoptotic, and lipid metabolism disorder regulation. In addition, studies have shown that BBR can regulate intestinal flora to maintain the stability of the intestinal environment. The various methods of BBR diabetes treatment are shown in Figure 1.

### 3.1. Antihyperglycemic

**Figure 2:**
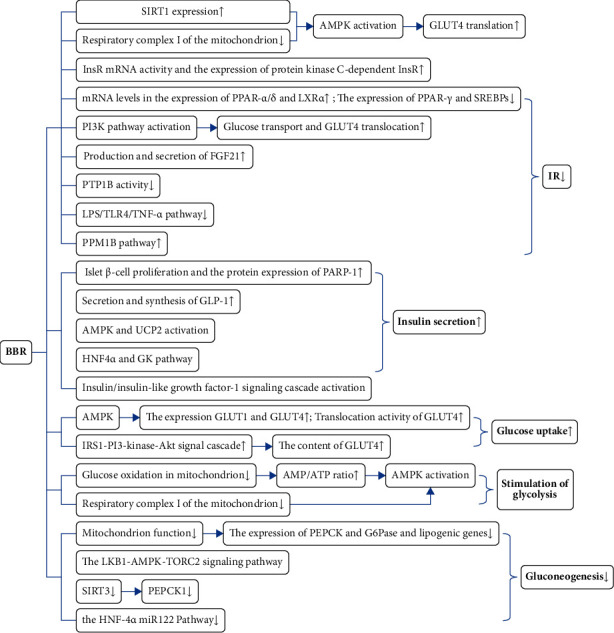
The main pathways and molecular mechanisms of BBR in lowering blood sugar (↑ = increase or improve; ↓ = decrease or inhibit).

Diabetes mellitus is a metabolic disease characterized by high blood sugar caused by defective insulin secretion or impaired insulin action. The prerequisite for diabetes treatment by lowering blood sugar is to understand the source and destination of blood sugar and how to regulate blood sugar concentration. BBR can weaken blood sugar source by inhibiting liver gluconeogenesis and strengthen glucose metabolism by inducing glycolysis. In addition, BBR can also regulate blood glucose concentration by enhancing insulin sensitivity, reducing IR, and promoting insulin secretion. The specific BBR-related pathways and molecular mechanisms in lowering blood sugar are shown in Figure 2. In addition, the animal and cell experiments of BBR's hypoglycemic effect are summarized in Table 2.

#### 3.1.1. Increasing Insulin Sensitivity and Alleviating IR

AMP-dependent protein kinase (AMPK) plays a vital role in the process of regulating the body's systemic energy homeostasis. BBR may enhance insulin sensitivity and reduce systemic obesity to maintain glucose homeostasis through AMPK activation in multiple tissues including muscles and adipose tissue [67]. For instance, BBR promoted glucose transporter-4 (GLUT4) transport to the plasma membrane and increased insulin sensitivity of insulin-resistant H9c2 cardiomyocytes by activating AMPK [68]. Two main BBR mechanisms were noted to activate the AMPK pathway. One is that BBR inhibits respiratory complex I of the mitochondrion, thereby stimulating the AMPK activity [19, 65]. The other is that BBR upregulated the expression of sirtuin 1 (SIRT1) in adipose tissue so that the AMPK pathway can be activated [69]. Eventually, the effects of inhibiting inflammation, ameliorating IR, and increasing insulin sensitivity are achieved.

The insulin receptor (InsR), a vital factor in the insulin signaling pathway, is extremely important for the physiological effects of insulin on cells [70]. BBR could increase the activity of InsR mRNA and upregulate protein kinase C-dependent InsR expression, thereby effectively increasing glucose utilization and improving IR [42–44]. Simultaneously, BBR had the ability to upregulate InsR gene expression muscle cells and in the liver in a protein kinase D- (PKD-) dependent manner, which contributed to promoting insulin sensitivity [71]. BBR behaved like an insulin sensitizer in these mechanisms to some extent.

In addition, experiments illustrated that BBR alleviated IR by regulating the expression of genes related to metabolism in the liver of diabetic rats. Specifically, BBR could not only upregulate the mRNA levels in the expression of the liver X receptor and peroxisome proliferator-activated receptors but also downregulate the expression of sterol-regulatory element-binding proteins and PPAR-*γ*, which improved fat-induced hepatic IR [45, 46]. Furthermore, BBR alleviated IR through fibroblast growth factor 21 (FGF21). FGF21 is a hormone derived from the liver that exerted important effects on regulating body metabolism. In IR mice, BBR could regulate glucose metabolism and increase insulin sensitivity through mechanisms such as inducing FGF21 production and secretion and promoting white fat browning [48, 72]. In the myotube IR model, BBR administration activated the phosphatidylinositol 3-kinase (PI3K) pathway, which in turn increased GLUT4 translocation and acute insulin-mediated glucose transport, thus alleviating IR [47].

BBR inhibited the protein tyrosine phosphatase 1B activity of mice embryonic fibroblasts 3T3-L1 adipocytes in a dose-dependent manner, thereby improving IR [49]. In addition, BBR inhibited the lipopolysaccharide (LPS)/toll-like receptor 4 (TLR4)/tumor necrosis factor-*α* (TNF-*α*) pathway, suggesting that this pathway may be one of the BBR mechanisms to lower blood glucose and moderate IR [50]. Moreover, Wu et al. demonstrated that BBR may alleviate IR via regulation on the protein phosphatase, Mg^2+^/Mn^2+^-dependent 1B (PPM1B) signaling pathway, including the PPM1B/inhibitor kappa B kinase*β* (IKK*β*)/nuclear factor *κ*B (NF-*κ*B), and PPM1B/GLUT4 pathways [51].

#### 3.1.2. Promoting Insulin Secretion

BBR can promote insulin secretion with a hypoglycemic effect by pancreatic *β*-cells [73]. The main mechanism is discussed in the following sentences. Moreover, BBR may stimulate insulin secretion mainly through improving PARP-1 protein expression and pancreatic *β*-cells proliferation [52]. Besides, as a hormone secreted by the intestinal tract, glucagon-like peptide-1 (GLP-1) has a variety of physiological functions, such as stimulating insulin secretion, promoting the proliferation of pancreatic *β*-cells, and regulating glucose metabolism. Furthermore, BBR could increase the secretion and synthesis of GLP-1 when blood sugar is elevated, thereby promoting insulin secretion in cells and regulating insulin level in the body [53].

Furthermore, uncoupling protein 2 (UCP2) has the function of regulating glucose-stimulated insulin secretion (GSIS). BBR enhances insulin secretion in the pancreatic islets of diabetic mice and HG-treated rat insulinoma cell lines via the activation of UCP2 and AMPK pathways [54]. BBR probably causes pancreatic *β*-cell proliferation and GSIS through an enhanced insulin/insulin-like growth factor-1 signaling cascade and pathways related to hepatocyte nuclear factor 4*α* and glucokinase [55, 74].

However, some experiments found that BBR has an inhibitory effect on insulin secretion in a dose-dependent manner and significantly weakened insulin secretion at high glucose concentrations. Research on the mechanism of action in this area is relatively scarce. One possible mechanism is BBR inhibited GSIS via the KATP-dependent triggering pathway [56].

#### 3.1.3. Promoting Glucose Uptake

Glucose relies on glucose transporters to enter the cell after glucose is absorbed into the blood. GLUT1 is expressed in all human tissues, and GLUT4 is mainly expressed in insulin-sensitive myocardium, skeletal muscle, fat cells, and myocardium. BBR can activate GLUT1 and upregulate GLUT1 expression level through the AMPK pathway and increase GLUT4 expression and translocation activity, thereby enhancing the glucose uptake and elevating the glucose availability by tissues and cells of the body [57–59]. For example, BBR enhanced the IRS1-PI3-kinase-Akt signal cascade of adipocytes to increase GLUT4 content in the membrane, which was beneficial for glucose uptake promotion [74]. BBR also had a mechanism of promoting glucose uptake through the AMP-AMPK-p38 mitogen-activated protein kinase (MAPK) pathway rather than the insulin signaling pathways. Thus, BBR may stimulate glucose uptake and consumption via increased the production and accumulation of triacylglycerol and decrease cellular diacylglycerol (DAG) levels in H9c2 cells [60].

#### 3.1.4. Inducing Glycolysis

AMPK was the main energy sensor, which was turned on when the glucose level rose and turned off when the glucose level fell. Experiments revealed that the BBR effect on lowering blood sugar did not necessarily depend on AMPK activation. In other words, BBR played a bidirectional role in regulating AMPK phosphorylation. Moreover, when the blood sugar rose in vivo, BBR played a beneficial role in stimulating glycolysis by inhibiting glucose oxidation in the mitochondria, which was associated with the inhibition of glucose oxidation in the mitochondria. Thus, AMPK activation may be caused by the increase in the AMP/ATP ratio due to mitochondrial inhibition [61]. In contrast, other scholars found that BBR stimulated glycolysis and improved sugar metabolism via inhibiting mitochondrial respiratory chain complex I, which did not depend on AMPK activation, in case of nonhypoglycemia [62, 63].

#### 3.1.5. Inhibiting Gluconeogenesis in the Liver

Glucose-6-phosphatase (G6 Pase) and phosphoenolpyruvate carboxykinase (PEPCK) were two key rate-limiting enzymes that regulated hepatic gluconeogenesis through their transcriptional modulation [64]. BBR may inhibit mitochondrial function, and the expression of PEPCK, G6 Pase, and adipogenic genes that depend on ATP for energy supply is reduced, thereby inhibiting gluconeogenesis. Through inhibiting SIRT3, BBR could cause PEPCK1 instability to make the development of gluconeogenesis in the liver to be attenuated [59]. Beyond that, BBR inhibited liver gluconeogenesis in diabetic rats through the liver kinase B1-AMPK transcription coactivator 2 signaling pathway [53, 54]. BBR suppressed the HNF-4*α* miR122 pathway in type 2 diabetic mice, which contributed to the attenuation of gluconeogenesis and lipid metabolism disorder [66].

### 3.2. Antioxidant Stress and Anti-Inflammatory Response

**Figure 3:**
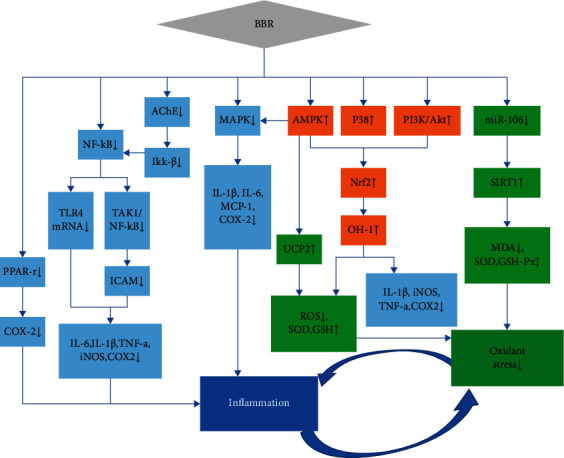
Mechanistic aspects of the antioxidant and anti-inflammatory action of BBR (↑ = increase or improve; ↓ = decrease or inhibit; the blue rectangle represents the related pathway mechanism of inflammatory response; the green rectangle represents the related pathway mechanism of oxidative stress; and the orange color represents the related pathway mechanism of inflammatory response and oxidative stress).

Current studies have reported that BBR has a therapeutic effect on type 2 diabetes mellitus (T2DM) and its complications by affecting inflammation and oxidative stress. Numerous pieces in the literature showed that inflammation and oxidative stress had a close relationship. They have several common signal pathways. The vicious circle of the two will further cause IR. Similarly, the imbalance of the intestinal flora in diabetic patients will also lead to low-grade inflammation, oxidative stress, IR, and vice versa. In various tissues and cells related to metabolism, berberine could play an anti-inflammatory role through reducing the expression of key proinflammatory cytokines interleukin-6 (IL-6), interleukin-1*β* (IL-1*β*), and TNF-*α*. Similarly, research results confirmed that BBR exerted an indispensable influence on antioxidative stress by regulating antioxidant enzymes and oxidative stress markers [75]. BBR acted against oxidative stress and inflammation via an extremely complex mechanism consisting of some signaling pathways (including NF-*κ*B, AMPK, nuclear factor erythroid-2-related factor-2 (Nrf2)/oxygenase, and MAPKs pathway), as well as various kinases in cells [76, 77]. The available data on BBR administration have revealed the close connection between oxidative stress and inflammation and different cellular pathways as illustrated in Figure 3. The animal and cell experiments of BBR administration that act against oxidative stress and inflammation are shown in Table 3.

As a kind of transcription factor, Nrf2 can stimulate the transcription of antioxidant- and anti-inflammatory-related genes. Moreover, PI3K/Akt, AMPK, and P38 signaling pathways all participated in the BBR-mediated antioxidant and anti-inflammatory responses. Through these signaling pathways, BBR activated the nuclear Nrf2 pathway so that it exerts many effects, including upregulating antioxidant enzyme superoxide dismutase (SOD), glutathione peptide (GSH), and glutathione peroxidase (GSH-Px) and inhibiting the generation of reactive oxygen species (ROS) and the expression of inflammation-related factors such as IL-1*β*, IL-6, TNF-*a*, cyclooxygenase-2 (COX2), and inducible nitric oxide synthase (iNOS), which contributed to the BBR antioxidant and anti-inflammatory activities [77, 81]. Increasing pieces of evidence currently proved that heme oxygenase-1 (HO-1), an antioxidant enzyme whose expression is regulated by Nrf2, is a vital molecule. BBR could induce HO-1activation and exert comprehensive benefits on extenuating inflammation and oxidative stress [86].

Meanwhile, NF-*κ*B was also a transcription factor that promoted the expression of inflammatory factors. BBR inhibited the transforming growth factor beta-activated kinase 1/NF-*κ*B molecular signaling pathway via blocking the MAPK pathway and SIRT1-dependent mechanism and inhibited NF-*κ*B phosphorylation, which led to attenuating secretion and IL-1*β*, IL-6, iNOS, COX2, and TNF-*a* expression and decreasing the ICAM-1 expression, thereby suppressing inflammation [59, 82, 83]. In addition, BBR could inhibit the IKK/NF-*κ*B pathway and decrease the expression and secretion of inflammatory factors by inhibiting the activity of acetylcholinesterase in the cell supernatant. Except this, BBR could reduce TLR4 expression at mRNA and protein levels and the NF-*κ*B activity, which resulted in anti-inflammatory effects [84]. Furthermore, BBR suppressed the transcription and expression of COX2 and AP-1 through attenuating PPAR-*γ* expression and phosphorylation [77].

Previous experiments have verified that BBR directly or indirectly inhibited MAPK in a way that is dependent or independent of AMPK activation in macrophages as well as decreased the expression of proinflammatory factors IL-1*β*, IL-6, monocyte chemoattractant protein-1, and COX2 [77, 82, 83, 85]. BBR could not only increase GSH, SOD, and GSH-Px contents but also decrease the marker of oxidative stress malondialdehyde (MDA) content. The regulation of these factors was beneficial in dealing with the problem of excessive free radicals in the oxidative stress response [76, 78, 79]. BBR ameliorated oxidative stress through increasing UCP2 and SOD, decreasing ROS and MDA production, and decreasing reduced coenzyme II NADPH oxidase expression. These may be mediated via the mitochondrial SirT3 pathway, miR-106b/SIRT1, SIRT1/FOXO (the forkhead box O), or AMPK pathway [77, 80].

### 3.3. Regulating the Lipid Metabolism Disorder

Fat accumulation plays a significant role in the occurrence and development of IR. Numerous experiments have confirmed that BBR had a pivotal role in regulating adipose tissues. BBR could significantly promote the conversion of liver cholesterol and the formation of bile acids and then stimulate its rapid excretion to bile to prevent lipid accumulation and rerelease of free fatty acids. Thus, the levels of low-density lipoprotein cholesterol (LDL-C), triglycerides (TGs), and serum total cholesterol significantly decreased and high-density liptein cholesterol and NO levels significantly increased, suggesting that lipid catabolism was promoted [87–91]. By stimulating the low-density lipoprotein receptor (LDLR) expression activated by extracellular regulated protein kinases (ERK), BBR could stabilize low-density lipoprotein receptor (LDLR) mRNA, thus regulating the level of LDL-C [89, 90].

Furthermore, PPARs regulate the fat metabolism and fat differentiation processes as the main transcription factor of fat metabolism and differentiation. Experiments indicated that PPAR*γ* mRNA expression is significantly reduced after BBR administration, thereby inhibiting the PPAR*γ* transcriptional activity and inhibiting fat production. To be specific, BBR increased the PPAR *α*/*δ* and ATP binding cassette transporter A1 levels; downregulated the PPAR *γ* and PPAR *γ* co-activator-1 levels; and activated the c-Jun N-terminal kinases, AMPK-p38 MAPK-GLUT4, and PPARs pathways to moderate lipid metabolism [45, 92, 93]. The NR activity in the feces could be used as a biomarker for personalized hyperlipidemia treatment using BBR. According to reports, the NR of the intestinal bacteria is a key factor to promote BBR intestinal absorption. Also, the regulation of the NR activity in the intestine by BBR may be one of its lipid-lowering mechanisms [94].

### 3.4. Regulating Gut Microbiota

The ecological balance of the intestinal flora will have a significant impact on the therapeutic effect of drugs. Drug prototypes and their metabolites will have a certain impact on the number and abundance of the intestinal flora, resulting in changes in intestinal homeostasis, thus affecting disease occurrence and development [95]. Current studies have shown that metabolic syndrome is closely related to the role of intestinal flora, and abnormal metabolism of substances mediated by intestinal flora is a significant pathological mechanism of metabolic syndrome. Due to the low BBR bioavailability, the classical pharmacokinetic theory is not enough to fully explain its hypoglycemic effect. Therefore, many scholars believe that the BBR hypoglycemic effect is not produced after absorption, but plays a role in the intestine [33]. Chen et al. [96] showed that BBR did have the ability to regulate the intestinal flora, protect the intestinal mucosal barrier, resist inflammation, and inhibit glucose absorption. Moreover, Xu et al. demonstrated that BBR treatment significantly altered the overall intestinal microbiota structure and enriched many butyrate-producing bacteria, thereby reducing blood sugar levels and alleviating inflammation [97]. Through the regulation of these florae, the levels of blood lipids and blood sugar in patients with metabolic diseases can improve, and it has a positive effect on the treatment of obesity, hypertension, T2DM, and other diseases.

The intestinal microecological imbalance in patients with T2DM is mainly manifested by changes in the composition, abundance, and function of the intestinal flora. More than 90% of the human intestinal flora are phylum Firmicutes and Bacteroidetes, which can be divided into three categories: beneficial bacteria (*Bifidobacterium*, *Bacteroides*, *Lactobacillus*, *Blautia*, and so on), conditional pathogenic bacteria (*Enterococcus*, *Escherichia coli*, and so on), and harmful bacteria (*Staphylococcus*, *Proteus*, and so on) [98, 99]. Among them, *Bifidobacterium* and *Lactobacillus* can improve the level of inflammatory factors. In addition, blood lipids can also be reduced and antihypertensive substances (e.g., angiotensin-converting enzyme inhibitory peptides), which can directly or indirectly reduce blood pressure and improve the level of blood lipids in patients, can be produced. Firmicutes can increase the absorption of carbohydrates and fat in the human intestine (called *fat bacteria*), while the role of *Bacteroides* is just the opposite (called *lean bacteria*). The decrease of the ratio of Firmicutes/*Bacteroides* can inhibit the growth of *Enterococcus faecalis* in the intestinal tract and simultaneously promote the growth of beneficial bacteria and suppress the incidence of obesity. After BBR treatment, the abundance of *Blautia* and *Bacteroides* increased and the abundance of *Faecalibacterium prausnitzii* and *Escherichia* flora decreased. Meanwhile, the ratio of Bacteroides/Firmicutes restoration resulted in decreasing levels of blood sugar and blood lipids, as well as IR improvement [87, 100–102].

BBR mainly affects the short-chain fatty acid metabolism, bile acid metabolism of the intestinal flora, and chronic low-grade inflammation caused by increased LPS for diabetes treatment. Through certain metabolic pathways, the intestinal microflora can produce short-chain fatty acids, which can protect the intestinal mucosal barrier, regulate host energy intake and metabolism, reduce the level of blood glucose and blood lipids, alleviate chronic low-grade inflammation, and inhibit endotoxins [103, 104]. Animal experiments have proved that the intestinal flora produces butyrate and the content of butyrate in plasma or feces was significantly increased after BBR treatment, confirming that butyrate was an active endogenous metabolite that cooperated with BBR to lower blood lipids [75, 105]. BBR promoted the production of butyric acid bacteria through the acetyl-CoA-butyryl-CoA-butyrate pathway and enables it to synthesize butyrate, and butyrate then entered the blood, thus decreasing blood sugar and blood lipids [97, 104, 106].

Experiment results demonstrated that BBR could improve the intestinal microenvironment, thereby protecting the intestinal barrier function, which may be achieved by regulating intestinal microorganisms [87]. In the diabetic rat model, BBR treatment is efficient in improving endotoxemia, restoring intestinal permeability, and repairing the damaged intestinal mucosa and systemic inflammation by regulating intestinal microflora. Meanwhile, these effects of increasing endocrine regulatory peptide (peptide, PYY), regulating the GLP-1 and GLP-2 secretions, promoting the differentiation of L cells in the colon, and improving the intestinal peptide level in rats fed with a high-fat diet all resulted from BBR [107–109]. Farnesol X receptor (FXR) activation had a pretty favorable effect on the treatment of hyperlipidemia, hyperglycemia, and diabetes [110–112]. Moreover, Sun et al. proved that BBR could activate the FXR signal and directly affect intestinal flora to change bile acid metabolism, thereby improving diabetes and its complications [113].

## 4. The Interaction between Drugs

Li et al. proposed that the BBR pharmacological effects in vivo weakened after separation and purification. This may be caused by the difference in pharmacokinetic characteristics between purified BBR and BBR in *Rhizoma Coptidis* after intestinal absorption [114]. The difference in BBR pharmacokinetics in vivo may be related to the interaction with other components and drugs. The interaction between them is mainly manifested in various ways discussed in the subsections below.

### 4.1. P-Glycoprotein

The P-gp substrate is affected by the P-gp efflux. Experiments confirmed that BBR was indeed the P-gp substrate. Due to the P-gp efflux, the BBR concentration in the cell had significantly decreased [115, 116]. The solution to this problem was the synergistic use of P-gp inhibitors and BBR to reduce the BBR efflux and improve BBR absorption [5]. For example, the combination with P-gp inhibitors (e.g., tetrandrine) could enhance the hypoglycemic effect, thereby dramatically improving the pharmacokinetics [22]. The P-gp inhibitor D-*α*-tocopherol polyethylene glycol-1000 succinate can significantly promote BBR absorption, which contributed to improving poor BBR bioavailability [23]. A Caco-2 cell monolayer test indicated that glycyrrhizic acid could promote BBR permeability through inhibiting P-gp [24]. In addition, the P-gp inhibitor cyclosporine A reduced the BBR efflux rate by more than 30 times [25]. Similarly, in in vitro experiments, oligomeric proanthocyanidins significantly reduced the P-gp expression, increased the average maximum BBR concentration, and promoted the BBR uptake. This led to improving pharmacokinetics in vivo [26].

### 4.2. Cytochrome P450

CYP450 could metabolize a large number of pharmaceutical ingredients with different structures, especially those with low oral bioavailability. BBR was a substrate of CYP450, and its metabolic process was affected by CYP450. CYP450 inhibition could lead to the interaction of drugs, and the CYP450 activity seems to depend on the BBR dose administered. The experiment proved that CYP2D and CYP1A2 played a vital role in the BBR biotransformation. Giving high BBR doses may inhibit the CYP1A2 activity and interfere with the metabolism of other drugs [32, 39]. In other words, the metabolic BBR kinetics combined with other drugs was largely affected by the CYP450 activity [117].

### 4.3. Organic Cation Transporter and Multidrug and Toxin Efflux

BBR is not only a P-gp substrate but also an organic cation transporter (OCT) 2 and multidrug and toxin efflux (MATE) 1 substrate. Moreover, MATE1 had a significant impact on BBR distribution and excretion. It affected BBR distribution in the liver and its bile excretion. Meanwhile, it affected BBR distribution in the kidneys and its excretion to the feces. In addition, MATE1 mediated BBR renal carrier transport. As a specific MATE inhibitor, pyrimethamine could inhibit BBR outflow at high concentrations, thereby increasing BBR concentration in cells. Similarly, OCT2 mediated BBR renal carrier transport. As an OCT2 inhibitor, corticosterone could inhibit BBR efflux at high concentrations [118, 119]. Experimental studies have found that, in the absence of inhibitors (e.g., pyrimethamine and corticosterone), BBR could induce pharmacokinetic interactions by combining with other drugs to inhibit OCT- and MATE1-mediated transport [120, 121].

### 4.4. Combination with Other Antidiabetic Western Medicines

Western medicines (e.g., metformin, glipizide, pioglitazone, glibenclamide, sitagliptin, and so on) are all good hypoglycemic drugs. However, the application of western medicine alone has limited therapeutic effects on diabetes. Thus, combining with BBR is a way to enhance the effect. For example, the combined use of BBR and metformin could competitively inhibit organic cation transporters on the cell membrane, increase the absorption of metformin, and better exert its blood-sugar-lowering effect [120]. The BBR and sitagliptin combination significantly reduced blood sugar and blood lipids and inhibited the secretion of proinflammatory factors [122]. The BBR and glibenclamide combination improves the MDA level better than using it alone, thereby improving oxidative stress [123].

## 5. Security

According to literature analysis, the occurrence of toxic and adverse reactions was related to the BBR dose. Almost no toxic and side effects were noted at common doses, and only few patients had mild gastrointestinal reactions, including diarrhea and constipation [124–126]. Some experiments have calculated the BBR lethal dose after administration. The measured 50% lethal dose (LD50) and the level of adverse reactions vary greatly depending on the way and the subject of BBR administration. No LD50 was found in the oral administration group. Meanwhile, the LD50 values in the BBR intraperitoneal and intravenous injection were 9.04 and 57.61 mg/kg, respectively. In mice and rats, the lowest levels of BBR maternal toxicity were 841 and 531 mg/kg/day, respectively. Current data proved that no obvious side effects were noted when BBR was used in combination with other drugs [127–129].

## 6. Conclusions and Future Recommendation

It is worth noting that the effects of berberine in lowering blood sugar, antioxidative stress, and anti-inflammatory response; regulating lipid metabolism; and regulating intestinal flora are not separated but mutually influence and promote each other. Regarding the pharmacokinetic BBR studies, more studies are based on animal experiments, and fewer reports were noted in humans because its pharmacokinetic behavior is unsatisfactory. By analyzing BBR pharmacokinetic absorption, distribution, metabolism, and excretion, the problem of low BBR bioavailability was noted, which is currently the main reason for restricting the BBR application. Fortunately, continuously increasing studies dedicated to solving the poor BBR absorption exists. The article mainly enumerates three methods: altering the delivery system, adjusting the chemical structure, and using it in coadministration with P-gp inhibitors. Regarding BBR metabolism, most studies have shown that it is often metabolized into other products. However, its pharmacological effects may vary after being metabolized into other products. Therefore, how to discover the active BBR metabolites and moderately increase the oral bioavailability of berberine to maximize the effectiveness of the drug will be the focus of follow-up studies.

The BBR mechanism in diabetes treatment has been extensively discussed, mainly focusing on the five aspects of lowering blood sugar, anti-inflammatory, and antioxidative stress and regulating lipid metabolism and intestinal flora. Berberine is involved in these five aspects. The roles of the parties are not separate but mutually influence and promote each other. The activation of some pathways may play multiple roles. For example, the AMPK activation and the SIRT1 expression promotion does not only lower blood sugar but also serve as anti-inflammatory and antioxidative stress. Intestinal flora has attracted increasing attention in recent years. Studies on the correlation between inflammation, oxidative stress, and intestinal flora in diabetes are also increasing. Moreover, the imbalance of intestinal flora in diabetic patients can also lead to low-grade inflammation, oxidative stress, IR, and vice versa. The summary of the BBR antidiabetic mechanism and the summary of pharmacokinetics can provide more ideas for clinical application studies. These studies can support the necessity of long-term BBR use in diabetes treatment and prevention and its complications and have important reference value and preventive measures for clinical medicine. However, through the summary of the mechanism of action, the molecular mechanism of BBR's antidiabetic is still not deep enough. Some pathways are only possibly effective, and no definite proof has been noted. Therefore, more detailed and in-depth studies are needed in the future.

As for the interaction between BBR and drugs, few studies exist, especially in combination with Western medicine. Most of the studies separately compare the BBR hypoglycemic effects and Western medicines. Some studies have found that the combination of sugar and Western medicine is better than single use. Thus, a combination of the two to improve diabetes is a direction worth studying. Moreover, intestinal flora has attracted increasing attention in recent years. However, studies on BBR regulation of dysbacteriosis and diabetes prevention are not thorough and systematic, and the specific mechanism and material basis are unclear. Studies in this area are more limited to pharmacodynamic indicators. In addition, the effective pathways and targets are unclear. Therefore, more attention should be focused on the mechanism in the future. Multigroup combination techniques (e.g., metabonomics, macrogenomics, and proteomics) can be used to understand the pathogenesis of the disease from the perspective of the overall metabolism, clarify the specific targets and pathways of BBR intervention, and further verify it at the gene and protein levels. Based on the current study status, it is recommended that the changes in the intestinal flora after BBR intervention should be carried out in depth and systematically, and the correlation with diabetes should be analyzed in depth so that finding active ingredients or component groups with definite effects and clear mechanisms would become easier.

## Figures and Tables

**Table 1:** Summary of ways and examples to improve the bioavailability of BBR.

Methods	Examples	Effect	References
Alternative delivery system	Dendrimer encapsulated and conjugated delivery of BBR	Improved BBR oral bioavailability	[9]
	Clear anhydrous reverse micelles containing amorphous BBR nanoparticles	Enhanced the hypoglycemic effect	[10]
	BBR nanosuspension	Improved lipid metabolism, lowered blood sugar	[11]
	Selenium-coated nanostructured lipid carriers	Increased intestinal absorption, improved the hypoglycemic effect	[12]
	Polymer-lipid hybrid nanoparticles loaded with BBR-phospholipid complex	Enhanced the oral efficiency, improved sustained release	[13]
	BBR-loaded solid lipid nanoparticles	Strengthened intestinal absorption, enhanced the antidiabetic effect	[14]
	A novel BBR-loaded cremochylomicron	Improved BBR oral bioavailability	[15]

Chemical structure modification	The mannose modified BBR derivative	Increased antidiabetic activity	[16]
	Synthesis of disaccharide modified BBR derivatives		[17]
	BBR derivative: nandinine	Inhibited inflammation, attenuated IR	[18]
	BBR derivative: dihydroberberine	Enhanced insulin sensitivity, attenuated IR	[19]
	9-O (lipophilic group substituted) BBR derivatives	Increased hypoglycemic activity	[20]
	C-9 modified BBR derivatives	Improved lipid-lowering activity	[21]

Coadministration with P-gp inhibitors	Tetrandrine	Decreased the efflux rate of BBR, promoted intestinal absorption	[22]
	D-*α*-tocopheryl polyethylene glycol 1000 succinate (TPGS)		[23]
	Glycine (GLY)		[24]
	Cyclosporine A (CsA)		[25]
	Oligomeric proanthocyanidins (OPCs)		[26]

**Table 2:** Effects of BBR on antihyperglycemic in animals or cells with diabetes mellitus.

Effects of BBR	Animal model and cells type	Dosage and usage of BBR	References
Increasing insulin sensitivity and alleviating IR	HFD mice and rats	In vivo, 100 mg/kg/d (p.o. for 2 weeks)	[19]
	HFD mice with or without Sirt1 knockout	In vivo, 20 or 50 mg/kg/d (p.o. for 2 weeks)	[42]
	HFD + STZ T2DM rats; HepG2 cells	In vivo, 75 or 150 mg/kg twice a day (p.o.); in vitro, 1 nmol/L of insulin or 7.5 *μ*g/ml of BBR	[43]
	HFD + STZ T2DM rats	In vivo, 200 mg/kg/d (p.o.)	[44]
	STZ T2DM rats	In vivo, 75, 150, 300 mg/kg/d (p.o. for 16 weeks)	[45]
	HFD + STZ T2DM hamsters	In vivo, 150 mg/kg/d (p.o. for 9 weeks)	[46]
	L6myc cells	7.5 *μ*g/ml of BBR (for 1 h)	[47]
	HFHS fed mice with SIRT1 LKO	In vivo, 5 mg/kg/d (i.p. for 5 weeks)	[48]
	db/db mice and DIO mice; 3T3-L1 adipocytes L6 myocytes	In vivo, 100 mg/kg/d (p.o. for 2 weeks); in vitro, 1.25 *μ*M and 1.5 *μ*M; in vitro, 2.5–5 *μ*M	[49]
	HFD and SPF rats	In vivo, 100 mg/kg/d (i.p. for 8 weeks)	[50]
	HFD and ZDF rats	In vivo, 100 or 300 mg/kg/d (i.p. for 12 weeks)	[51]

Promoting insulin secretion	HFD + STZ T2DM rats	In vivo, 100 mg/kg/d (i.p. for 8 weeks)	[52]
	Rats fed with commercial stock diet; human NCI-H716 cells	In vivo, 60 or 120 mg/kg/d (p.o. for 5 weeks); in vitro, 0, 1, 10 and 100 *μ*M	[53]
	Rats insulinoma INS-1E cells with HG	In vitro, 0, 1, 5 and 10 *μ*mol/L	[54]
	Rat pancreatic islets	In vitro, 1, 3, 10 and 30 *μ*mol/L	[55]
	Rat islets	In vitro, 0, 0.5, 2.5, 5 and 10 *μ*M for 1 h	[56]

Promoting glucose uptake	3T3-L1 fibroblasts	In vitro, 1–10 *μ*M for 6 h	[57]
	HFHS diabetic hamsters	In vivo, 50 or 100 mg/kg/d (p.o. for 6 weeks)	[58]
	293 T cells and primary hepatocytes	In vitro, 0, 1, 5 and 10 *μ*M	[59]
	H9c2 cardiomyoblast cells	In vitro, 12.5 *μ*M for 24 h	[60]

Inducing glycolysis	HFD rats; 3T3-L1 preadipocytes and myoblasts L6	In vivo, 125 mg/kg twice a day (i.p. for 5 weeks); in vitro, 0, 2 and 10 *μ*mol/L	[61]
	HepG2 and mouse skeletal myoblast C2C12	In vitro, 0, 2, 5, 10 and 20 *μ*M	[62]
	HepG2 hepatocytes and C2C12 myotubes	In vitro, 10 *μ*M	[63]

Inhibiting gluconeogenesis in the liver	HFD + STZ diabetic rats	In vivo, 156 mg/kg/d (i.p. for 12 weeks)	[64]
	HFD + STZ diabetic rats	In vivo, 380 mg/kg/d (p.o. for 5 weeks)	[65]
	HFD + STZ diabetic rats	In vivo, 40 or 160 mg/kg/d (p.o. for 4 weeks)	[66]

HFD: high-fat diet; HepG2: human hepatocellular carcinomas; SIRT1 LKO: liver-specific Sirt1 knockout; DIO: diet-induced obesity; HFHS: high fat, high sucrose; SPF: specific pathogen free; ZDF: Zucker diabetic fatty.

**Table 3:** Effects of BBR on oxidant stress and inflammation in animals or cells with diabetes mellitus.

Animal model, cell type	Samples examined	Dosage	Effects	References
ddY mice, STZ 100 mg/kg, single i.p. injection	Liver	200 mg/kg/d p.o. for 2 weeks	↓: GPx; ↑: SOD	[78]
ICR mice, nicotinamide 1000 mg/kg + STZ 100 mg/kg, single i.p. injection	Liver and kidney	100 mg/kg/d, p.o. for 2 weeks	↓: MDA; ↑: SOD	[79]
ICR male mice, STZ 30 mg/kg, single i.p. injection	Serum and islets	5 mg/kg/d, i.p. for 3 weeks	↓: MDA; ↑: SOD	[80]
Male Wistar rats, ATO (arsenic trioxide) 7 mg/kg, p.o.	Mitochondria and hepatocytes	100 mg/kg/d, p.o. for 8 days	↓: ROS	[81]
RAW 264.7 macrophages treated with or without LPS	264.7 macrophages	1–20 *μ*M pretreated for 4 hours before LPS treatment for 20 hours	↓: IL-6, TNF-*a*	[82]
Male albino Wistar rats, STZ 40 mg/kg, single i.p. injection	Serum and liver	BC 50 mg/kg/d, i.p. for 45 days	↓: TNF-*a*, COX-2, iNOS; ↑: SOD, GSH, GPx	[83]
RAW 264.7 macrophages treated with LPS	Culture media	BH 10, 20, and 40 *μ*M for 0–48 h.	↓: TNF-*a*, IL-6, IL-1*β*	[84]
RAW 264.7 macrophages treated with BBR or LPS ICR mice with or without Nrf2	Culture media Lung	5 *μ*M treated for 24 hours BBR mg/kg and LPS injection 15 mg/kg i.p. for 6 hours	↓: IL-6, COX-2, iNOS, ROS	[85]

↑ = increase or improve; ↓ = decrease or inhibit; BC: berberine chloride.
